# More than a pore: How voltage-gated calcium channels act on different levels of neuronal communication regulation

**DOI:** 10.1080/19336950.2021.1900024

**Published:** 2021-06-09

**Authors:** Jennifer Heck, Ana Carolina Palmeira Do Amaral, Stephan Weißbach, Abderazzaq El Khallouqi, Arthur Bikbaev, Martin Heine

**Affiliations:** a Functional Neurobiology, Johannes Gutenberg-University Mainz, Institute for Developmental Biology and Neurobiology, Mainz, Germany; b Computational Genomics and Bioinformatics, Johannes Gutenberg-University Mainz, University Medical Center Mainz, Institute for Human Genetics, Mainz, Germany

**Keywords:** Ion channels, voltage-gated calcium channels, VGCC auxiliary subunits, α_2_δ subunits, Ca_v_β subunits, synaptogenesis, gene regulation, synaptic transmission, voltage-induced calcium release, alternative splicing

## Abstract

Voltage-gated calcium channels (VGCCs) represent key regulators of the calcium influx through the plasma membrane of excitable cells, like neurons. Activated by the depolarization of the membrane, the opening of VGCCs induces very transient and local changes in the intracellular calcium concentration, known as calcium nanodomains, that in turn trigger calcium-dependent signaling cascades and the release of chemical neurotransmitters. Based on their central importance as concierges of excitation-secretion coupling and therefore neuronal communication, VGCCs have been studied in multiple aspects of neuronal function and malfunction. However, studies on molecular interaction partners and recent progress in omics technologies have extended the actual concept of these molecules. With this review, we want to illustrate some new perspectives of VGCCs reaching beyond their function as calcium-permeable pores in the plasma membrane. Therefore, we will discuss the relevance of VGCCs as voltage sensors in functional complexes with ryanodine receptors, channel-independent actions of auxiliary VGCC subunits, and provide an insight into how VGCCs even directly participate in gene regulation. Furthermore, we will illustrate how structural changes in the intracellular C-terminus of VGCCs generated by alternative splicing events might not only affect the biophysical channel characteristics but rather determine their molecular environment and downstream signaling pathways.

## Introduction

Transient changes of the intracellular calcium concentration are a major trigger for many signaling cascades and cellular processes. Besides being a key parameter for neuronal communication, intracellular calcium levels control the entire cell life from fertilization to programmed cell death and regulate, inter alia, gene expression, heart and muscle contraction, as well as enzyme activity within subcellular compartments [1–3]. In excitable cells like neurons, voltage-gated calcium channels (VGCCs) are important regulators of the calcium concentration by controlling the influx of calcium ions (Ca^2+^) across the plasma membrane [4–6]. The huge signaling power of Ca^2+^ , which is the most widely used and at the same time most strictly controlled second messenger molecule [1,7], might account for the fact that mutations in VGCCs have been reported in the context of severe disorders reaching from cardiovascular channelopathies to neurological and psychiatric conditions such as ataxic and epileptic phenotypes, chronic pain, autism, schizophrenia, and depression [8–10]. However, there is a growing body of evidence suggesting that the functional relevance of VGCCs goes beyond their central role as Ca^2+^ -conducting elements. One structural feature that might facilitate such a many-sided picture of VGCCs is their design as multi-subunit complexes. The basic Ca^2+^ -conducting pore is formed by the α_1_ subunit, a 190–270 kDa membrane-spanning protein. Today, ten variants of the pore forming α_1_ calcium channel subunits have been described and grouped into three families, termed Ca_V_1, Ca_V_2 and Ca_V_3, based on their biophysical kinetics and pharmacological properties [4,11]. This diversity of calcium channel phenotypes is strongly increased by the association of the auxiliary subunits β, α_2_δ, and γ as well as many other regulatory proteins that interact with specific binding domains located in the intracellular loops of α_1_[9,12]. Historically, the functional importance of auxiliary VGCC subunits was considered primarily in association with the α_1_ pore of Ca_V_1 and Ca_V_2 high-voltage-activated (HVA) VGCCs. From this point of view, β and α_2_δ isoforms were extensively shown to promote the trafficking of the channel to the membrane, as well as to significantly modulate the biophysical properties of the multi-subunit channel complex [13,14]. However, several reports published over the last two decades demonstrate that VGCC auxiliary subunits can serve additional functions, which do not necessarily involve or require a direct interaction with the channel pore. Furthermore, there are studies suggesting that the pore-forming subunit of VGCCs, besides being the traditional source of Ca^2+^ from the extracellular space, has some side-functions, e.g. as voltage sensor and interaction partner for signaling complexes as well as in gene expression. These extended roles of VGCCs will be discussed in the following to illustrate some possibilities of how VGCCs might participate in neuronal network development, maintenance, and plasticity.

## VGCCs: Not only voltage-gating but voltage-sensing

**Figure 1.**
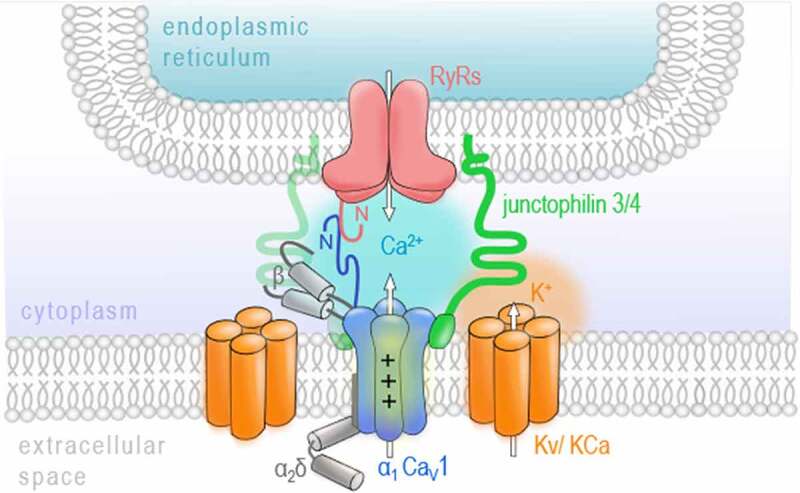
VGCCs as voltage sensors

As already mentioned above, VGCCs are key regulators of the Ca^2+^ influx across the plasma membrane of excitable cells. In neurons, they have been widely described to shape neuronal communication by initializing the release of neurotransmitter molecules. This process mainly involves the transient influx of Ca^2+^ in response to the depolarization of the plasma membrane triggered by an arriving action potential and is therefore known as excitation-secretion coupling [4–6]. However, besides the Ca^2+^-conducting aspect, a second key feature of VGCCs is their ability to sense membrane depolarization to initialize channel gating. The role VGCCs as voltage sensors was firstly described in skeletal muscle. Here, the excitation-contraction coupling does not require the influx of extracellular Ca^2+^ via VGCCs but rather depends on their voltage-sensing properties to trigger the release of Ca^2+^ from intracellular stores of the sarcoplasmic reticulum [15–17]. This functional coupling involves the physical interaction of Ca_V_1.1, a member of the Ca_V_1 family, and calcium release channels in the sarcoplasmic reticulum called ryanodine receptors (RyR), especially the isoform 1, called RyR1. When binding to the skeletal RyR1, Ca_V_1.1 transduces the sarcolemma depolarization to directly induce a mechanical gating of RyR1 by conformational interaction resulting in calcium release from intracellular stores [18,19]. Interestingly, the molecular basics and, more importantly, a similar process of voltage-induced calcium release (also called depolarization-induced calcium release) has been documented in the brain and spinal cord [20–23]. Mouton and colleagues have found RyR1 channels, although poorly expressed in the brain when compared to the other RyR isoforms 2 and 3 [23–27], in a complex with the pore-forming subunits of the Ca_V_1 family members Ca_V_1.2 and Ca_V_1.3 in the rat brain [22]. The mechanical coupling between RyR1 and Ca_V_1 channels was later shown to be specific for Ca_V_1.2, while RyR2 was associated with Ca_V_1.3 in spinal cord dorsal columns and whole brain [20]. A study by Kim et al. has further characterized the specific interaction of Ca_V_1.3 and RyR2 in the rat hippocampus and demonstrated the importance of both N termini of Ca_V_1.3 and RyR2 for their functional coupling [23]. Notably, also the activation of RyR2 in hippocampal neurons seems to be independent of the Ca^2+^ influx through Ca_V_1.3 since RyR2 opening was also triggered in Ca^2+^ -free extracellular solution [23]. This highlights the function of Ca_V_1.3 as a voltage sensor in neurons, which seems to differ from its classical role in the cardiac muscle where Ca_V_1.3 opening has been widely reported to trigger calcium-induced calcium release [28]. Although the co-localization of Cav1 isoforms and RyRs in neurons was confirmed using immunocytochemistry and super-resolution microscopy [23,29], it has been noted that only a few of the Ca_V_1-RyR complexes appear co-localized and are sparsely distributed along axon cylinders [20]. More recently, studies have revealed additional interaction partners, including potassium channels and junctophilin proteins, participating in a multi-protein complex on the junction between the outer plasma membrane and the ER in mammalian neurons (Fig.1) [29,30]. However, further investigation is necessary to evaluate the stability of these complexes and their functional implication for, e.g. synaptic plasticity. Suggestions have been made on a role in activity-dependent transport of signaling molecules [20], neuronal excitability [29], and a possible link to transmit membrane activity to gene expression in the nucleus [22]. From a pathophysiological perspective, it has been shown that RyR1 mediates the release of damaging quantities of Ca^2+^ from the ER when triggered by ischemic depolarizations sensed by Ca_V_1.2 in rat dorsal columns [20]. Therefore, the voltage-sensing contribution of VGCCs in the release of Ca^2+^ from intracellular stores should be considered when studying pathophysiological elevations of calcium levels involved in, e.g. axonal damage [31]. Although a participation of the Ca^2+^ influx through VGCCs cannot be ruled out completely, especially for Ca_V_1.3-RyR2-complexes, this functional coupling is a first example of how VGCCs, as calcium sensors and interaction partners, can contribute to more complex signaling mechanisms.

As known from the skeletal muscle, also in neurons VGCCs have been shown to colocalize with ryanodine receptors (RyR) in the endoplasmic reticulum and trigger RyR-mediated Ca^2+^release from intracellular stores. Importantly, the RyR opening, especially of RyR isoform 1, was shown to be independent of the ionic activity of the VGCCs but rather require the conformational change of the voltage-sensing transmembrane helices of the VGCC pore induced by membrane depolarization, a process called voltage-induced calcium release. Additional interaction partners like voltage-gated (Kv) or Ca^2+^-activated (KCa) potassium channel and membrane-binding proteins, junctophilins, might complement and stabilize this functional complex.

## C-terminal fragments of the VGCC pore and Ca_V_β subunits participate in gene regulation

**Figure 2.**
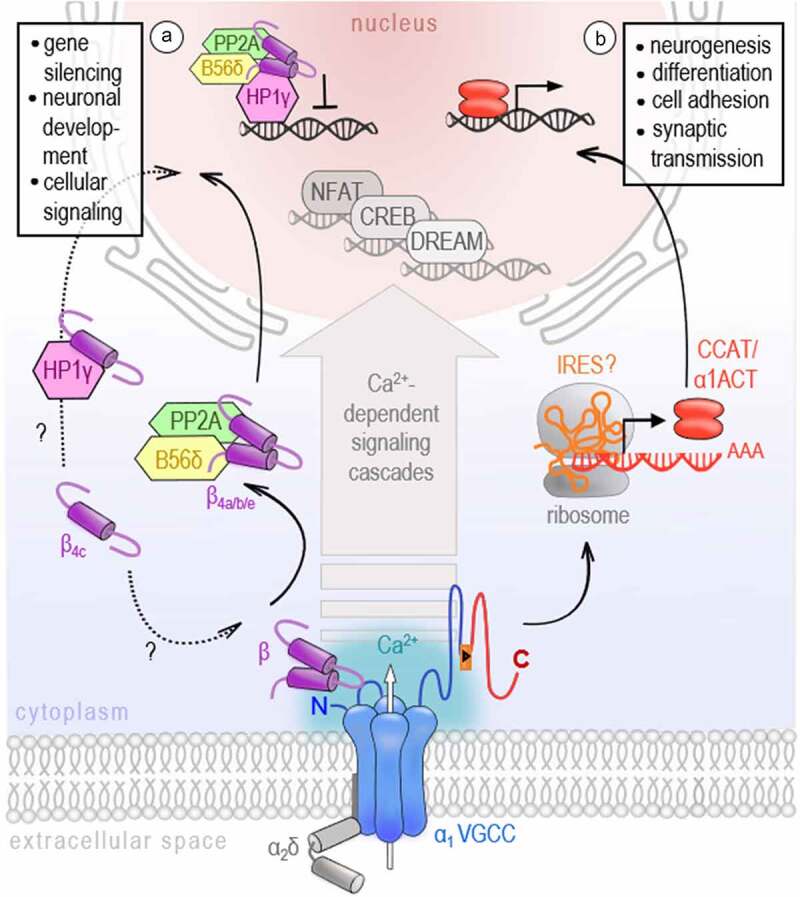
VGCCs participate in gene regulation

Since intracellular Ca^2+^ acts as a second messenger molecule in a plethora of signaling pathways [1], it is evident that VGCCs,  beeing main plasma membrane Ca^2+^ sources in excitable cells, contribute to transcriptional regulation processes [32]. This excitation-transcription coupling allows the conversion of activity-induced, very local calcium transients into long-term effects on gene regulation pathways with distinct transcription factors. Early studies have already demonstrated that the genes regulated via VGCC-induced Ca^2+^ influx differ from those targeted by other Ca^2+^ origins, for example, receptor-activated Ca^2+^ channels like NMDA receptors, store-operated Ca^2+^ channels like ORAI and TRP members, or by Ca^2+^ release from intracellular stores [33,34]. To date, many pathways orchestrating neuronal development, survival, and communication have been identified that specifically involve VGCCs' Ca^2+^ signaling to activate CREB [35,36], NFAT [37] or downstream regulator element antagonist modulator (DREAM) transcription factors [38], to name only a few [32]. Even if the specific contribution of VGCCs has been reported to initialize these pathways, it is the transcriptional signaling power of Ca^2+^ in combination with accessory Ca^2+^-binding proteins that allows for the nuclear forwarding and transcriptional action described above. However, several observations have been made, underpinning the idea that VGCC subunits and intracellular domains might also directly act as gene regulators (Fig.2).

Besides Ca^2+^-dependent signaling cascades activating NFAT, CREB, or DREAM, VGCC β_4_ subunits (A, left) and α_1_ C-terminal domains (B, right) also act as transcription factors. A) β_4_ splice variants interact with proteins of the epigenetic machinery such as HP1s or the B56δ regulatory subunit of protein phosphatases-2A (PP2A) and translocate to the nucleus with a rank order of ᵝ_4b_ > ᵝ_4a_ > ᵝ_4e_. B) CCAT and ɑ_1_ACT are fragments directly derived from the carboxyl-terminus of the pore-forming α_1_ subunit of Ca_V_1.2 and Ca_V_2.1 channels, respectively. Their expression via an internal ribosomal entry site (IRES) or an unknown exonic promoter is controversially discussed. They have been shown to translocate to the nucleus and regulate gene activity associated with neurodevelopment and synaptic transmission. Modelled after Barbado et al. 2009^32^ and Tadmouri et al. 2012^57^.

In 2006, two independent studies have reported C-terminal fragments of Ca_V_1.2 and Ca_V_2.1 that translocate to the nucleus of neurons in vitro and in vivo [39,40]. Gomez-Ospina et al. describe a so-called calcium channel associated transcriptional regulator (CCAT), a C-terminal fragment of the Ca_V_1.2 channel subunit. Initially, it was assumed that CCAT is proteolytically processed from the full-length Ca_V_1.2. However, later studies have shown that an internal promoter located in exon 46 of the *CACNA1C* gene might drive CCAT’s expression independently of the Ca_V_1.2 channel protein [41]. CCAT was verified in many different neuronal cell types throughout the brain, with a strong nuclear expression in GAD65-positive inhibitory neurons [39,41]. Notably, its nuclear localization is developmentally regulated and controlled by changes in intracellular calcium levels [39]. In the nucleus, CCAT associates with the transcriptional regulator p54(nrb)/NonO [39] and can activate transcription reporters and endogenous genes affecting, for example, cell excitability, neurite extension, and neural differentiation [42]. A similar observation has been made by Kordasiewicz and colleagues, who have found a C-terminal fragment of the neuronal Ca_V_2.1 VGCC, which they have termed α_1_ACT, enriched in nuclei of cerebellar neurons [40]. The expression of α_1_ACT was proven to involve the cap-independent translation as a second gene product via an internal ribosomal entry site (IRES) located in the C-terminus of the *CACNA1A* gene [43]. Using chromatin immunoprecipitation-based sequencing and high-throughput RNA sequencing (RNA-seq), α_1_ACT was shown to orchestrate a complex network of neuronal genes associated with neurogenesis (*Dusp4, Efnb2, Fgfr3, Gfra2, Ntn1, Ptger3, Penk*, and *Odc1*), synaptic transmission (*Hcn4, Slc18A3*, and *Syn2*), and cell adhesion (*L1cam*) essential for early cerebellar development and neonatal survival [43,44]. Tet-off transgenic reintroduction of α_1_ACT in Purkinje cells of knockin-knockout compound heterozygote mice (KIKO), which exhibit a marked reduction of the full-length Ca_V_2.1 mRNA isoform (+exon47), improved mouse survival and early motor development demonstrating a highly age-dependent operation window of α_1_ACT in early life [44]. Importantly, α_1_ACT was further shown to partially rescue the *CACNA1A* knockout phenotype associated with seizures, dystonia, ataxia, and death by postnatal days P18–P21 [45], at behavioral, histological, and electrophysiological levels [44]. These results demonstrate that both gene products, the ion channel and a nuclear protein α_1_ACT, play an important role in neuronal development and that in the case of perinatally decreased *CACNA1A* gene expression, the reintroduction of α_1_ACT might be a potential early intervention therapy [44]. Taken together with the results from Ca_V_1.2 and recent reports on C-terminal proteins identified across all functional VGCC classes (also Ca_V_1.3 [46] and Ca_V_3.2 [44]), it seems likely that the bicistronic expression of calcium channel proteins might be conserved across the gene family, even if a nuclear protein has not been documented for all channel isoforms, yet, and the nature of its expression mechanism is currently not fully elucidated.

Notably, not only intracellular domains of the VGCC pore have been identified to participate in transcriptional regulation, but also some isoforms of the auxiliary Ca_V_β subunit of the VGCC complex have been found in the nucleus. This intracellular family of VGCC subunits is widely known to support the trafficking and expression of functional VGCCs. Four isoforms of the Ca_V_β subunit are described (β_1_-β_4_), each having different splice variants that might interact with any α_1_ channel isoform in a tissue-dependent manner [11,47]. In addition to their essential effect on the forward trafficking of VGCCs [48,49], β subunits can also modulate the channel’s kinetics, reported as a shift of the activation potential and raise of the opening probability of HVA channels resulting in larger current densities [14,50,51]. More surprisingly, several studies uncovered a calcium channel-independent function of β_4_ which has been shown to translocate to the nucleus and might be directly involved in activity-dependent gene regulation. At first, Hibino and colleagues have described an atypical short splice variant of the β_4_ subunit, namely β_4C_, to directly interact with the chromo shadow domain of chromobox protein 2/heterochromatin protein 1γ (CHCB2/HP1γ), a nuclear protein involved in the epigenetic control of gene regulation and gene silencing. While having only slight effects on channel activation and inactivation kinetics, the co-expression of β_4C_ with CHCB2/HP1γ fosters the recruitment of β_4C_ to the nuclei of mammalian cells and significantly reduces its transcriptional repression activity [52]. Despite some controversial results about the molecular underpinnings, a number of studies have now confirmed the nuclear targeting of various β_4_ variants [52–55] and their direct involvement in gene regulation via interactions with proteins of the epigenetic machinery such as HP1s [52,56] or the regulatory subunit of protein phosphatases-2A [57,58]. Notably, the subcellular localization and thus the function of β_4_, either as a VGCC channel subunit or transcription regulator, was shown to be under the control of electrical activity and Ca^2+^ influx [54,57]. Subramanyam et al. further interpret this activity-dependent shuttling of β_4b_ into and out of the nucleus, and probably its switch between two independent physiological functions, as a possible mechanism of VGCCs to communicate their state of activity to the nucleus. Reconstitution experiments performed on cultured hippocampal neurons and cerebellar granule cells prepared from E17 lethargic (β_4_-null) mice have shown that the extent of β_4_ nuclear targeting significantly varied for the tested β_4_ splice variants which localized in neuronal nuclei with a rank order of β_4b_ > β_4a_ > β_4e_ [55]. The differential subcellular distribution of β_4_ splice variants suggests that they might regulate distinct genetic programs. Indeed, Etemad and colleagues report that the gene regulatory power of β_4_ splice variants correlates with their nuclear-targeting properties. However, they further point out that the nature of regulated genes, which are mainly implicated in cellular signaling, membrane/vesicle transport, and neuronal development, is quite similar for the tested β_4_ splice variants [55]. This could indeed indicate an identical gene regulatory mechanism but remains to be explored in more detail.

## α2δ subunits are many-sided extracellular interaction partners involved in synaptogenesis

**Figure 3.**
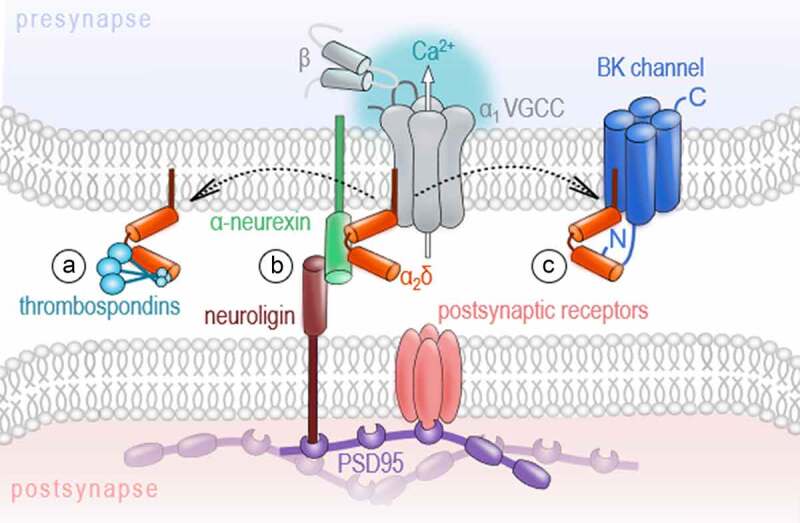
Channel-independent functions of the auxiliary ɑ_2_ δ subunit

In the extracellular space, VGCCs are represented by the auxiliary α_2_δ subunits that are attached to the outer loops of the α_1_ subunits. To date, four α_2_δ isoforms (α_2_δ_1_-α_2_δ_4_), encoded by the *CACNA2D1-CACNA2D4* genes, have been identified [13,14]. Being transcribed from a single gene [59], the α_2_δ protein undergoes post-translational proteolytic cleavage into α_2_ and δ polypeptides [60] that remain linked via disulfide bonds [61]. Although it is generally assumed that every α_2_δ isoform can associate with any HVA α_1_ pore-forming subunit, recent findings suggest distinct α_1_-preferences for some α_2_δ isoforms [62–65]. Hence, the observed differences in the distribution and expression levels of the individual α_2_δ isoforms across tissues and brain regions [66–71] might reflect such preferential interaction between α_2_δ and α_1_ subunits. Experimental and clinical data on α_2_δ knockouts or mutations in α_2_δ genes revealed their important role for the development of neuronal networks and establishment of excitation-to-inhibition balance. In particular, genetic aberrations in *CACNA2D1* and *CACNA2D2* in humans are associated with a developmental delay, mental disability and symptomatic epilepsy [72,73]. In several studies of normotypic and autistic individuals, mutations in *CACNA2D3* were consistently identified as a risk factor for autism spectrum disorders [74]. In the last few years, several new interaction partners of α_2_δ subunits were identified, including thrombospondin [75], prion protein [76], LRP1 [77], BK channels [78], NMDA receptors [79], and α-neurexin [62,63]. Given the wide range of possible interactions, the idea that α_2_δ subunits can exert calcium channel-independent functions is increasingly gaining favor (Fig.3)

A) Excitatory and inhibitory synaptogenesis: As interaction partners of thrombospondins, ɑ_2_δs were shown to foster glutamatergic synapse formation. Further, ɑ_2_δ subunits participate in GABAergic synaptogenesis. B) Transsynaptic signaling: ɑ_2_δ subunits interact with α-neurexin, which is involved in the formation of transsynaptic complexes supporting synapse specification, establishment, maturation, and plasticity. C) Competition of other interaction partners, like the large conductance (big) potassium (BK) ɑ subunit, for ɑ_2_δ.

.

One of the most intriguing aspects related to such autonomous functions of α_2_δ subunits is their ability to trigger synaptogenesis in developing neurons. The α_2_δ_1_ subunit was shown to induce glutamatergic synapse formation in murine neurons through a process requiring interaction with thrombospondins, extracellular matrix proteins released by young astrocytes [75]. Similarly, *straightjacket* (the ortholog of α_2_δ_3_) was reported to be essential for the development of excitatory synaptic boutons in *Drosophila*, with the extracellular α_2_ peptide chain being necessary and sufficient for bouton formation [80]. The finding of Kurshan and colleagues that the synapse establishment was significantly impaired upon deletion of *straightjacket*, but was not affected by the knockout of the pore-forming subunit *cacophony* [80], provided one of the first pieces of evidence that synaptogenic function of α_2_δ subunits is independent of the α_1_ subunit. A more recent report provided further compelling evidence for the channel-independent action of α_2_δ subunits on the formation of presynaptic release machinery and transsynaptic organization. In cultured hippocampal neurons and at the calyx of Held, triple knockout of Ca_V_2.1, Ca_V_2.2, and Ca_V_2.3 strongly impaired evoked vesicle release but did not alter the structure of presynaptic terminals or transsynaptic organization [81]. Importantly, the localization of the α_2_δ_1_ in nerve terminals was not affected by the knockout of Ca_V_2 channels as compared to control neurons, hence demonstrating that the synaptic localization of the α_2_δ_1_ is independent of the channel pore [81]. Furthermore, the total deletion of α_2_δ subunits in hippocampal neurons supported their relevance as a nucleation point for the formation of glutamatergic synapses [82]. Consistent with the role of α_2_δ_1_ subunits in glutamatergic synaptogenesis, the overexpression of α_2_δ_1_ in adult mice leads to cortical hyperexcitability, epileptiform activity, and an increased glutamatergic synaptic density [83], while a significant decrease in the number of excitatory synapses was shown for cortical neurons of α_2_δ_1_ knockout mice [84]. We have recently demonstrated that α_2_δ subunits can trigger the formation of not only excitatory, but also inhibitory synapses in an isoform-specific manner [64]. While α_2_δ_1_ subunits selectively improved neurotransmitter release in glutamatergic synapses, the upregulation of the α_2_δ_3_, but not α_2_δ_1_, resulted in a significantly higher density of GABAergic synapses and facilitated both the axonal growth in GAD67-positive interneurons and spontaneous GABA release during early development [64]. Although modulatory effects on the channel pore cannot be completely ruled out, the expression, surface delivery, and major current properties of Ca_V_2.1 and Ca_V_2.2 channels were not affected by association with either α_2_δ_1_ or α_2_δ_3_ in heterologous expression systems [64].

A strong argument for the autonomous function of α_2_δ subunits is the plurality of their additional interaction partners. At the nanoscale organization of a synapse, their interaction of α_2_δ subunits with the synaptic cell adhesion molecule α-neurexin might be particularly interesting in the context of transcellular units, so-called nanocolumns [85,86]. These structures have been recently reported to align the presynaptic nerve terminal, synaptic cleft, and postsynaptic compartment fostering efficient neurotransmission [85,86]. Similarly to α_2_δ subunits, α-neurexins exhibit a wide array of potential binding partners, including the postsynaptic neuroligin [87,88], neurexophilin [89], dystroglycan [90], LRRTM proteins [91,92], and cerebellin [93]. The complex formed by neurexin isoforms and neuroligins, for example, has been described as a transsynaptic assembly found at excitatory and inhibitory synapses that is involved in synapse specification, establishment, maturation, and plasticity [94]. Notably, Tong and colleagues have reported a selective interaction of the α_2_δ_3_ protein with neurexin1α[62]. Since the confirmation of a specific association between α_2_δ isoforms and α-neurexin failed in overexpression experiments [63], the physiological interaction could be regulated rather via a cell type- or synapse-specific expression of these molecules than selective binding mechanisms. It is also conceivable that slight differences in the binding affinities can dynamically influence these molecular interactions. This could transiently modulate the calcium channel properties and, in turn, synaptic transmission and plasticity in an activity- or synapse-specific manner. Indeed, other partners, besides α-neurexin, e.g. BK potassium channels [80], have been also described to compete with the channel pore for the α_2_δ subunits [78]. Moreover, the channel-to-α_2_δ coupling is expected to be rather loose [95–97] and the analysis of surface dynamics of the α_2_δ_1_ subunits and various α_1_ proteins further revealed, apart from a pool of α_2_δ_1_ bound to the channel, two subpopulations of the α_2_δ_1_ and α_1_ subunits that are not interacting with each other [98]. Whether the dissociation of the α_2_δ subunit from the channel pore located in the plasma membrane has direct consequences on the channel gating, stability or turn-over rates has been discussed in some studies but requires more attention to resolve the molecular mechanism [78,99,100]. Besides gating or stability effects on the channel, changes in channel positioning or in the multi-subunit complex organization could also be possible.

To summarize, the VGCC pore-forming subunit as well as the auxiliary β and α_2_δ subunits play an essential role in voltage-induced calcium release, gene regulation, neuronal synaptogenesis, and transsynaptic signaling, which do not necessarily involve the Ca^2+^ transients of the channel complexes. The modular expression as multi-subunit complexes provides a first basis for the diversity and functional range of these molecules. We aim to further develop the idea that the functional diversity of VGCCs could not only arise from different basic channel kinetic and ion-conducting properties but rather from variations in their interaction partners or molecular environment. Some interesting findings pointing in this direction come from studies of alternative splice variants of VGCCs, which will be in the focus of the last section of this review.

## Alternative splicing: How VGCC transcript variants shape neuronal phenotypes or vice versa?

**Figure 4.**
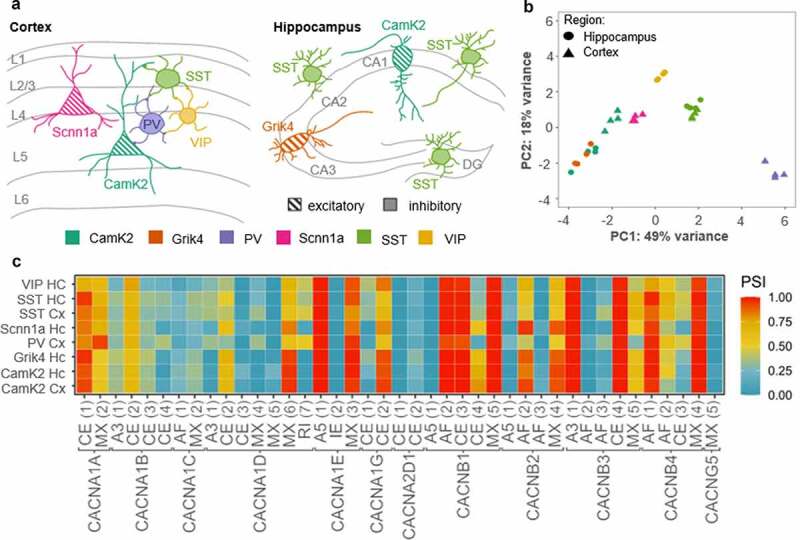
VGCC genes are spliced differently across neuronal cell types

Alternative splicing is crucial for increasing the proteomic diversity of the finite number of genes, which allows for a massive expansion of the coding power of the metazoan genome [101,102]. To explain the high functional diversity of neuronal phenotypes observed in distinct brain areas and over development, many studies have focused on the regulatory role of alternative splicing for molecules involved in neurotransmission, including large multi exon ion channels and postsynaptic receptors [103–108]. Nowadays, genome-wide analysis of splicing events strongly supports the idea of specialized splicing patterns in distinct neuronal phenotypes [109–111]. In the case of VGCCs, nine of the ten genes that encode the pore-forming α_1_ calcium channel subunit are expressed in the mammalian brain, and each α_1_ subunit contains multiple sites that are hotspots for splicing events [103,112]. However, whether VGCCs are indeed spliced in a cell type-specific manner has not been comprehensively assessed, yet. For a first estimation, we have analyzed raw sequencing data generated by Furlanis and colleagues (accession code: GSE133291 [110]; Fig. 4) from ribosome-associated transcript isoforms in genetically defined neuron types of the mouse forebrain. The data indicate that some classes of neurons indeed stand out for the splicing events of VGCCs, annotated to date. Especially the class of parvalbumin (PV)-positive cells, which have been described as fast-spiking GABAergic interneurons [113], show a distinct VGCC splicing pattern (Fig. 4C). Hence, there might be a link between neuronal cell identity and VGCC splicing. The structural changes induced by alternative splicing might affect channel gating and conductance, which then leads to different functional properties of neuronal phenotypes. There is an extensive literature accumulated over the last decades that clearly demonstrates major effects of VGCCs’ splicing on the channel’s pharmacology, gating, surface expression, but also on its molecular interactions [103,114,115]. Here, we want to present some examples, showing that alternative splicing has no direct consequence on the channel gating but rather affects their coupling to downstream interaction partners triggering different signaling pathways.

A) Schematic overview representing different neuronal cell populations included in the RNA sequencing data (data is publicly available; accession code: GSE133291^[110]^). The scheme was adapted from Furlanis and colleagues. The color-code for the cell types is used in B. CamK2= Ca^2+^/calmodulin-dependent protein kinase II; Grik4= glutamate receptor, ionotropic, kainite 4; PV= parvalbumin; Scnn1a=α subunit of the epithelial sodium channel ENaC; SSTsomatostatin; VIP- vasoactive intestinal peptide. B) The principal component analysis (PCA) of the gene expression demonstrates a differential regulation of VGCC genes across excitatory and inhibitory neuronal cell types. Especially, the cluster of PV interneurons localizes with asignificant gap to other neuron types on principal component 1, which explains roughly fifty percent of the observed variance. Data analysis: The fastq-files were mapped to the mm10 reference genome using STAR^[117]^. The PCA was performed with DESeq2 in R^[118,119]^ and plotted with ggplot2^[120]^. C) Heatmap of alternatively spliced transcripts of VGCC genes. We have analyzed the occurrence of the following splice events: cassette exon (CE), mutually exclusive exon (MX), retained intron (RI), alternative 5’ splice-site (A5), alternative 3’ splice-site (A3) and alternative first exon (AF), schematically shown in the lower part of C. Only those events that show a significant variance between the samples (ANOVA p-value < 0.01) were included. Overall, a difference between the PV neurons and the remaining samples in the splice patterns for the VGCC genes can be observed (e.g. *CACNA1A* MX(2), *CACNB1* CE(4) or *CANB3* MX (5)). Since the exon numbering depends on the transcript variant of a respective gene, the exact position for the splice variants (for instance, the start and end coordinates of the included exon) of the heatmap are listed here: *CACNA1A*: CE (1) chr8:84601756−84601821, MX (2) chr8:84614695−84614791, *CACNA1B*: A3 (1) chr2:4718122, CE (2) chr2:24642853−24642858, CE (3) chr2:24656711−24656722, CE (4) chr2:24682976−24683038, *CACNA1C*: AF (1) chr6:119196231−119196093, MX (2) chr6:118637730−118637813, *CACNA1D*: A3 (1) chr14:30129848, CE (2) chr14:30129789−30129848, CE (3) chr14:30137041−30137124, MX (4) chr14:30089296−30089379, MX (5) chr14:30107653−30107712, MX (6) chr14:30171296−30171399, RI (7) chr14:30128798−30129789, *CACNA1E*: A5 (1)chr1:154471338, CE (2) chr1:154404885−154405013, MX (3) chr1:154416061−154416157, *CACNA1G*: CE (1) chr11:94423671−94423724, CE (2) chr11:94439709−94439777,*CACNA2D1*: CE (1) chr5:16322539−16322595, CE (2) chr5:16341990−16342010, *CACNB1*:A5 (1) chr11:98010004, AF (2) chr11:98023034−98022887, CE (3) chr11:98010627−98010646, CE (4) chr11:98011343−98011497, MX (5) chr11:98010627−98010646, *CACNB2*: A5 (1) chr2:4971646, AF (2) chr2:4739216−14739763,AF (3) chr2:4763129−14763992, MX (4) chr2:14967942−14968075, *CACNB3*: A3 (1)chr15:98640686, AF (2) chr15:98631805−98631931, AF (3) chr15:98632376−98632520, CE (4) chr15:98640959−98640978, MX (5) chr15:98640959−98640978, *CACNB4*: AF (1)chr2:52676582−52676271, AF (2) chr2:52676831−52676271, CE (3) chr2:52556202−52556361, MX (4) chr2:52465894−52465913, *CACNG5*: AF (1) chr11:107915055−107914900. For this analysis, the fastq-files were mapped to the referencegenome (mm10) with Salmon^[121]^ and the percentage-spliced in (PSI) values were computedusing SUPPA2^[122]^.

One interesting VGCC splice event is the usage of two mutually exclusive exons 37a and 37b found in the C-terminus of the neuronal Ca_V_2 calcium channels (Ca_V_2.1 [123], Ca_V_2.2 [124] and Ca_V_2.3 [116]). Independent studies have shown that the splicing of exons 37a and 37b generates two variants, EFa (or 37a) or EFb (or 37b), of the EF-hand-like Ca^2+^-binding motif that acts as a molecular switch for calcium-dependent facilitation and might modulate different neuronal phenotypes in a spatiotemporal manner [105,123–127]. The recent analysis of RNA-seq data obtained from specialized neuronal cell types mapped the differential usage of exon37 for Ca_V_2.1 channels across excitatory and inhibitory neurons [111]. Huntley et al. confirmed that the mutually exclusive usage of exon37 correlates with opposite short-term plasticity behavior observed for principal excitatory neurons versus inhibitory cells (like PV interneurons). These results are in line with the electrophysiological characterization of rat hippocampal neurons where the expression of Ca_V_2.1[EFa] was shown to promote synaptic depression, while Ca_V_2.1[EFb] favored synaptic facilitation [105]. Thalhammer and colleagues have further demonstrated that Ca_V_2.1[EFa] is more tightly coupled to presynaptic scaffold proteins and the neurotransmitter release machinery when compared to Ca_V_2.1[EFb], which is characterized by a rather loose coupling [105]. Although a contribution of differing biophysical properties for Ca_V_2.1[EFa] and Ca_V_2.1[EFb] cannot be excluded, the authors pointed out that the variation of the synaptic efficacy between these splice variants is likely due to a differential organization and molecular coupling at the presynaptic site [105]. This suggests that the tighter coupling of Ca_V_2.1[EFa], preferentially expressed in PV neurons, might be necessary to define the PV phenotype, whereas excitatory neurons use the Ca_V_2.1[EFb] variant. As previously mentioned, the mutually exclusive usage of exon37 is conserved across neuronal Ca_V_2 channels [116,123,124]. For Ca_V_2.2 channels, it has been shown that alternative splicing of the exon37 plays an important role in voltage-independent inhibition via G proteins. The inclusion of exon37a results in the expression of a tyrosine residue (Y1747), which is absent in exon37b, that triggers a voltage-independent inhibitory pathway that increases the sensitivity of Ca_V_2.2 channels to opiates and the inhibitory neurotransmitter GABA [128]. In a following study, the Lipscombe laboratory has further demonstrated the importance of activity-independent inhibition of Ca_V_2.2 channels expressing exon37a for its function in nociceptors and morphine analgesia sensitivity *in vivo*, and thus the relevance for the pain pathway [129].

As already indicated above, the C-terminus of neuronal VGCCs has been described for its central role for the channel’s synaptic targeting and organization [108,130], interaction with scaffolding proteins [105,131–135], G protein signaling [128,129], gating [112,125,136–139], and consequently, for synaptic transmission and short-term plasticity[105,108]. Therefore, we now want to pay attention to another splice event in the distal part of the VGCC’s C-terminus that critically affects the length and binding sites expressed by this important structure. The expression of truncated VGCCs arising from alternative splicing events has been reported across neuronal calcium channel isoforms [114,140]. In the case of Ca_V_2.1 channels, a premature stop codon results in the expression of a channel variant lacking exon47, termed ∆47, that exhibits a 150–250 amino acids (depending on the species) shorter C-terminus compared to the fully expressed exon 47 (+47) [112,141]. This truncation was shown to affect the channel’s trafficking, its molecular arrangement, as well as synaptic transmission properties including short-term plasticity [108,142]. However, only mild effects on the channel kinetics have been reported so far [108,142]. Moreover, a study by Aikawa et al. using a knockin mouse line, which exclusively expresses Ca_V_2.1_Δ47_ (*CACNA1A*^CtmKO/CtmKO^), has shown that in cerebellar Purkinje cells, exon47 was not required to maintain such basic channel electrophysiological properties as current density-voltage relationships and channel inactivation. Instead, in the cerebellum, exon47 has a more important role in establishing channel interactions with scaffold proteins such as RIM-binding protein 2 and the auxiliary subunit β_4_. Surprisingly, the absence of exon47 did not only reduce interactions to scaffold proteins but also promoted the binding to GABA_B2_, a principal subunit of the G protein-coupled receptor for GABA, in the cerebellum of *CACNA1A*^CtmKO/CtmKO^ mice. This enhancement of Ca_V_2.1–GABA_B2_ interaction might contribute to the pathogenesis of absence seizures and motor incoordination observed in these animals [143–147].

These examples demonstrate that changes in the structure of VGCCs induced by splicing events also affect their interaction to partner molecules and suggest that significant functional implications cannot be reduced to their ionic activity.

## Outlook and perspectives

Although this review cannot fully capture the functional range of VGCCs, we aimed to draw attention to some side functions of VGCCs that go beyond their classical role as ion channels to induce local Ca^2+^ nanodomains necessary for neurotransmission or downstream Ca^2+^ signaling pathways. When associated with ryanodine receptors, VGCCs contribute to a functional complex to trigger voltage-induced Ca^2+^ release from intracellular stores, a process that is independent of their ionic activity. Further, VGCC auxiliary subunits as well as C-terminal domains of the α_1_ channel pore participate in gene regulation. Their activity- and Ca^2+^-dependent subcellular shuttling implicate a pathway how VGCCs communicate their own activity status to the nucleus and integrate transient Ca^2+^ signaling into longer-lasting transcriptional processes. These transcriptomic alterations should be considered especially when evaluating the phenotype of channelopathies, where most of the studies have primarily focused on explaining the pathological consequences based on the loss or gain of calcium channel function so far. Moreover, we have shown that α_2_δ auxiliary subunits are directly involved into neuronal network development and maintenance by fostering excitatory and inhibitory synaptogenesis and transsynaptic signaling. We have introduced some examples where structural changes in critical VGCC domains result in distinct synaptic plasticity behaviors and contribute, at least to some extent, to the specification of neuronal phenotypes. Notably, these changes in the channel structure do not necessarily involve differences in basic channel gating but might rather involve different protein environments or downstream pathways. Considering the many interaction partners of VGCCs, and from a molecular dynamics point of view, we want to point out the possibility that these large molecules could also serve as seeding points for molecular interactions. The question remains open at this point to what extent VGCCs can shape their environment by acting as a basic element bringing together important signaling molecules or how specific interaction partners that are present, e.g. in distinct neuronal phenotypes, regulate VGCCs’ functional diversity.

## Abbreviations

α_1_ACT: C-terminal fragment of the neuronal Ca_V_2.1 VGCC
A3: alternative 3ʹ splice site
A5: alternative 5ʹ splice site
AF: alternative 3ʹ splice site
BK: big potassium
Ca^2+^: calcium ions
CamK2: Ca^2+^/calmodulin-dependent protein kinase II
CCAT: calcium channel associated transcriptional regulator
CE: cassette exon
CHCB2/HP1γ: chromobox protein 2/heterochromatin protein 1γ
CREB: cAMP response element-binding protein
DREAM: downstream regulator element antagonist modulator
ER: endoplasmic reticulum
GABA: gamma-aminobutyric acid
GAD65/GAD67: glutamic acid decarboxylase 65/67
Grik4: glutamate receptor, ionotropic, kainate 4
HVA: high voltage-activated
IRES: internal ribosomal entry site
Kv: voltage-gated potassium channels
KCa: callcium-activated potassium channels
KIKO: knockin-knockout compound heterozygote mice
LRRM: leucine -rich repeat transmembrane proteins
mEPSCs: miniature excitatory postsynaptic currents
mIPSCs: miniature inhibitory postsynaptic currents
MX: mutually exclusive exon
NFAT: nuclear factor of activated T-cells
NMDA: N-methyl-D-aspartate
ORAI: calcium release-activated calcium channel protein
PCA: principal component analysis
PP2A: protein phosphatases 2A
PSI: percentage spliced in
PV: parvalbumin
RI: retained intron
RNA-seq: high-throughput RNA sequencing
RyR: ryanodine receptor
SCA6: spinocerebellar ataxia type 6
Scnn1a: α subunit of the epithelial sodium channel ENaC
SST: somatostatin
Tet: tetracycline
TRP: transient receptor potential channels
VGCC: voltage-gated calcium channel
VIP: vasoactive intestinal peptide
